# Chronic *in vivo* exposure to *Helicobacter pylori* VacA: Assessing the efficacy of automated and long-term intragastric toxin infusion

**DOI:** 10.1038/s41598-020-65787-3

**Published:** 2020-06-09

**Authors:** Robin L. Holland, Kristopher D. Bosi, Gregory H. Harpring, Jiayi Luo, Matthew Wallig, Heidi Phillips, Steven R. Blanke

**Affiliations:** 1 Department of Pathobiology, College of Veterinary Medicine, University of Illinois at Urbana-Champaign, Urbana, Illinois 61801 USA; 2 Department of Microbiology, School of Molecular and Cellular Biology, University of Illinois at Urbana-Champaign, Urbana, Illinois 61801 USA; 3 Department of Veterinary Clinical Medicine, College of Veterinary Medicine, University of Illinois at Urbana-Champaign, Urbana, Illinois 61801 USA; 4 Carle Illinois College of Medicine, University of Illinois at Urbana-Champaign, Urbana, Illinois 61801 USA

**Keywords:** Animal disease models, Bacterial toxins, Helicobacter pylori

## Abstract

*Helicobacter pylori* (*Hp*) secrete VacA, a diffusible pore-forming exotoxin that is epidemiologically linked to gastric disease in humans. *In vitro* studies indicate that VacA modulates gastric epithelial and immune cells, but the *in vivo* contributions of VacA as an important determinant of *Hp* colonization and chronic infection remain poorly understood. To identify perturbations in the stomachs of C57BL/6 or BALB/C mice that result specifically from extended VacA exposure, we evaluated the efficacy of administering purified toxin using automated infusion via surgically-implanted, intragastric catheters. At 3 and 30 days of interrupted infusion, VacA was detected in association with gastric glands. In contrast to previously-reported tissue damage resulting from short term exposure to *Hp* extracts administered by oral gavage, extended infusion of VacA did not damage stomach, esophageal, intestinal, or liver tissue. However, several alterations previously reported during *Hp* infection were detected in animals infused with VacA, including reduction of the gastric mucus layer, and increased vacuolation of parietal cells. VacA infusion invoked an immune response, as indicated by the detection of circulating VacA antibodies. These foundational studies support the use of VacA infusion for identifying gastric alterations that are unambiguously attributable to long-term exposure to toxin.

## Introduction

*Helicobacter pylori* (*Hp*) chronically infects half the world’s population, and is a major risk factor for the development of gastric ulcer disease and cancer^1–3^. *Hp* transmission and colonization, which occur at a high rate during early childhood^4–8^, results in a protracted inflammatory microenvironment within the infected glands of the stomach^9^. However, because subjects are often unaware of their infection status until the emergence of clinical disease, relatively little is known about the natural history of *Hp* infection over extended periods of time, including the contributions of individual virulence factors to bacterial persistence and disease progression.

The vacuolating cytotoxin (VacA) is the only known exotoxin secreted by *Hp*^10,11^. Although most *Hp* strains harbor the gene encoding VacA (*vacA*), extensive human epidemiological data indicate that *Hp* strains with alleles encoding VacA with high cytotoxic activity *in vitro*, are associated with increased severity of gastric disease^12–15^. In murine models, VacA is an important determinant of colonization^16–19^ and persistence^16^. Cell culture models have provided elegant insights into the consequences of VacA intoxication of epithelial cells, which include mitochondrial dysfunction and disruption of metabolic homeostasis^20,21^, the biogenesis of large intracellular vacuoles^22–24^, and, at higher concentrations, death by several distinct mechanisms^25–35^. However, understanding these *in vitro* observations in the context of chronic *Hp* infection has been challenging, in part, because the biodistribution of VacA in the stomachs of *Hp*-infected individuals is poorly understood. As a diffusible toxin within the extracellular environment, VacA can presumably access gastric cells both directly at and distal to the site of *Hp* colonization. However, proximal and distal effects of VacA within the stomach are likely to be different, based, in part, on the concentration gradient of toxin established by the diffusion of following secretion into the extracellular environment. Notably, higher concentrations of VacA are required for toxin-dependent cell death, than for toxin-mediated vacuole biogenesis or disruption of metabolic homeostasis^20,21,36^. In addition, at the site of *Hp* microcolony formation, VacA cellular activity is antagonized by CagA, an *Hp* effector injected directly into host cells via type IV secretion^37–44^.

Previous *in vivo* studies of VacA action have involved the administration of a single or several doses of VacA-containing *Hp* extracts by oral gavage into mice, resulting in damage to gastric tissue, including loss of gland architecture, erosion, ulceration of the gastric epithelium, and infiltration of inflammatory cells within the lamina propria^45–49^. Although straightforward, delivery of toxin by oral gavage does not faithfully recapitulate gastric exposure to VacA during chronic *Hp* infection, in part, because the perpetual emptying of stomach contents pre-empts the study of chronic toxin exposure^50,51^. With the goal of overcoming this limitation, we assessed the efficacy of intragastric infusion to administer VacA, for extended and uninterrupted periods of time, directly into the stomach lumen of C57BL/6 and BALB/C mice.

## Results

### Intragastric catheter implantation

The *in vivo* consequences of long-term exposure to VacA, which is secreted by *Hp* into the extracellular environment as a diffusible exotoxin during infection, are poorly understood. In this study, we examined the efficacy of infusing toxin, for periods of 3 or 30 days, into the stomachs of 5–6 week old, male and female C57BL/6 mice, which have been used as an animal model for studying *Hp* infection biology^52–55^. Prior to infusion, a sterile catheter (Fig. S1) was surgically implanted into the stomach of each animal, and attached to the forestomach serosa (Fig. S2). The free end of the catheter was tunneled under the skin and exteriorized on the back of the mouse, just caudal to the neck, providing a direct, unobstructed access port to the stomach (Fig. S2).

**Figure 1:**
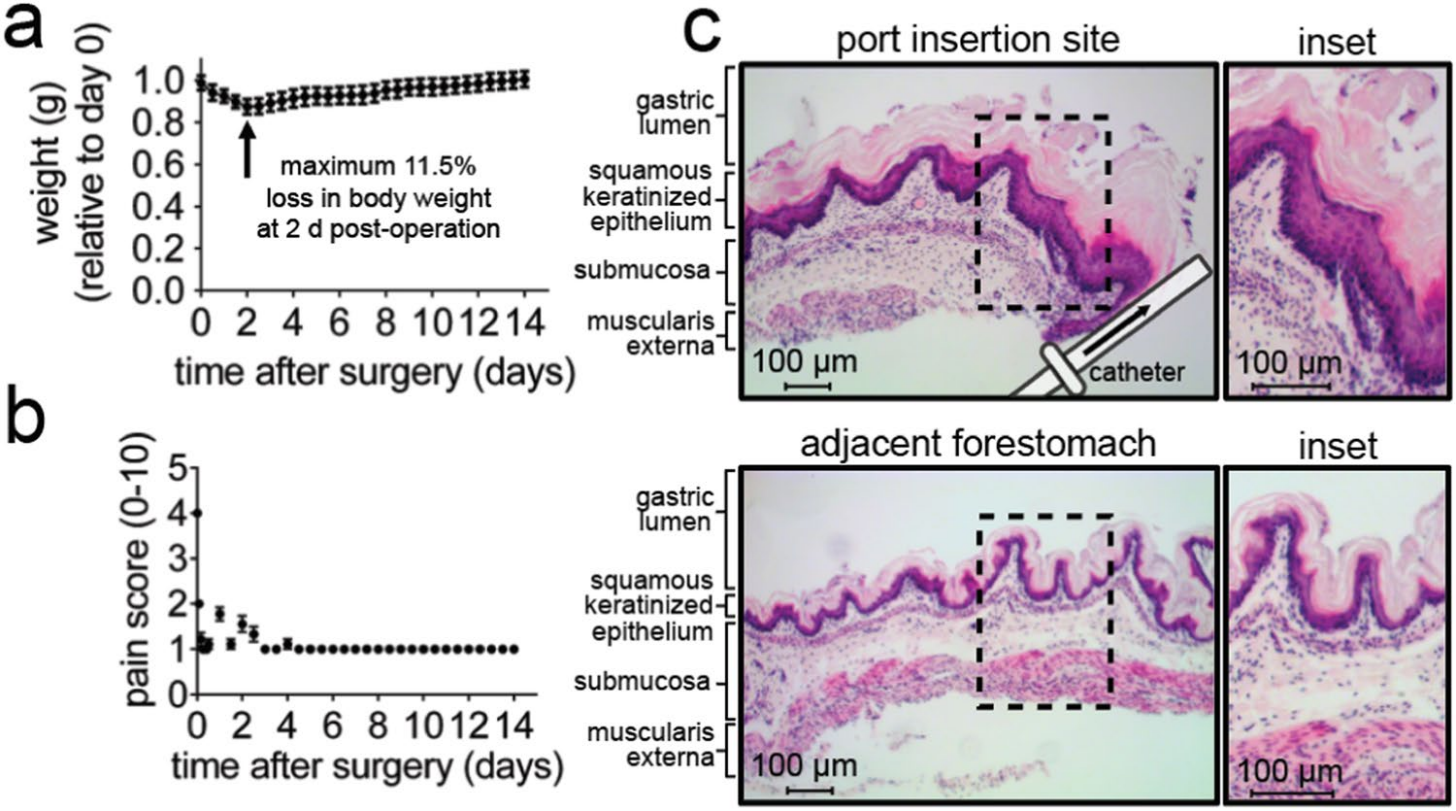
Post-surgical monitoring of animals following intragastric catheter implantation. (**a,b**) For the first 12 h following surgical catheter implantation, C57BL/6 mice were monitored every 2 h for (**a**) body weight and (**b**) pain on a scale of 0–10 (as evaluated by diminished mobility, grooming, and response to stimuli), and then thereafter every 12 h for 2 weeks. The data were combined from three independent surgical cohorts (n = 3), each performed in triplicate (9 animals total). Error bars represent standard error of the mean. (**c**) 2 weeks after surgical implantation of intragastric catheters, animals were euthanized, tissues were collected, paraffin embedded, and stained with hematoxylin and eosin (H&E). The site of catheter placement into the forestomach was observed by light microscopy imaging of a cross-sectional, 5 μm thick section. Images are representative of three independent surgical cohorts (n = 3) each performed in triplicate (9 animals total).

Following surgical catheter-implantation, animals were evaluated once every 2 h for the first 12 h, and then, once every 12 h for 2 weeks. Immediately following surgery, all animals exhibited visible and quantifiable stress, as indicated by a loss in body weight (Fig. 1a) and behavioral changes associated with pain (sickness behavior), including visible alterations in movement, grooming, and responses to stimuli (Fig. 1b). However, all behavioral modifications resolved within 5 days after surgery, and within 10 days, all animals had regained their original body weight. Based on these results, a two-week convalescent period between surgery and the commencement of infusion studies was adopted as standard practice.

### Intragastric infusion of VacA

C57BL/6 mice were administered, by gastric infusion, either purified VacA in saline solution, or saline solution alone (as the vehicle control), both of which had undergone acid-activation, as described under Materials and Methods. Infusion lines connected Luer ports, that had been surgically placed on the dorsum of each animal, to closed reservoirs containing the infusion solution (Fig. S3). Toxin preparations were refreshed within the infusion reservoirs every 24 h. Preliminary observations revealed that, after 1 day within closed infusion reservoirs, VacA retained greater than 50% of the toxin’s cellular activity (as measured by neutral red uptake), and between 25–50% of the toxin’s original cellular activity after 7 days. Because earlier papers had reported extensive and rapid gastric damage to the mice of mice administered single doses of VacA-containing bacterial extracts, we did not know prior to beginning these studies whether mice would tolerate long-term, uninterrupted toxin infusion. Rather than risk the unnecessary use of animals to establish a dose response curve for an untested system, we chose instead to examine a single concentration of toxin (500 nM), which under *in vitro* conditions, robustly causes cellular changes, including vacuolation, mitochondrial dysfunction, and autophagy induction^20,21,31,36,56–60^.

The volume of toxin or vehicle infused was controlled by a peristaltic pump interfaced with a dedicated computer (Fig. S4). The infusion lines were situated to allow animals unimpeded movement, but without the opportunity to interfere with the tubing. VacA or saline vehicle was infused at rates and frequencies estimated to maintain approximately 95% or 50% gastric capacities for 3-day and 30-day periods, which we refer to as short- or long-term exposures, respectively (Table 1). At the end of each infusion period, animals were euthanized by CO_2_ asphyxiation, followed by cervical dislocation. During the course of this study, none of the animals demonstrated detectable signs of stress or sickness. Moreover, system failures, such as leaks within the infusion lines, pump malfunctions, *etc*., were not encountered.

**Table 1:** Intragastric infusion parameters^*a*^.

infusion^b^	target stomach capacity^c^	experimental endpoint^d^	initial bolus^e^	initial bolus administration time^f^	re-infusion volume^g^	re-infusion adminitration time^h^	interval between re-infusions^i^
short-term	95%	12h	200 µl	5 min	50 µl	1 min	30 min
		1 day					
		3 days					
long-term	50%	5 days	100 µl	1 min	50 µl	1 min	45 min
		15 days					
		30 days					

^a^Animals were exposed to saline or saline-containing purified VacA under either a short-term infusion, with the stomachs filled to approximately 95% capacity, by administering an initial bolus of 200 μl over 5 min, then re-infusion of 50 μl over 1 min, every 30 min, for up to 3 days; or a long-term infusion, with the stomachs filled to approximately 50% capacity, by administering an initial bolus of 100 μl over 1 min, then re-infusion of 50 μl over 1 min, every 45 min, for up to 30 days.

^b^Infusions were designated as either short- or long-term.

^c^The percentage of the stomach volume targeted with each re-infusion.

^d^The time after the start of infusion when animals were euthanized and tissues collected.

^e^The volume of infusion solution administered initially in order to fill the stomach at approximately either 95% or 50% capacity, based on mouse cadaver studies.

^f^The duration of time that the initial bolus was administered over.

^g^The volume of infusion solution administered in order to fill the stomach at approximately either 95% or 50% capacity.

^h^The duration of time that the re-infusion solution was administered over.

^i^The time interval between the end of one infusion and the start of a re-infusion.

### Detection of VacA within the stomachs of toxin-infused animals

**Figure 2:**
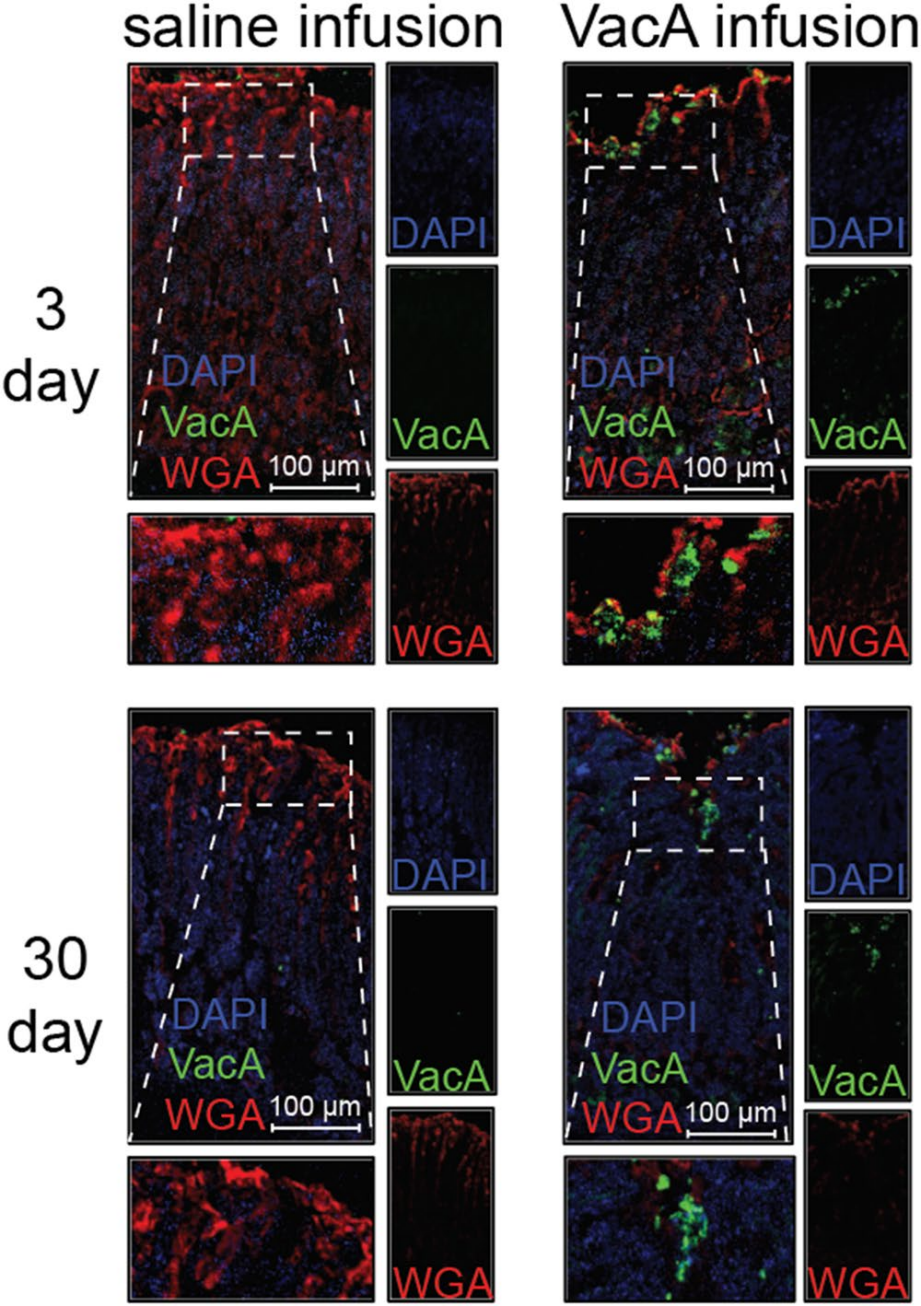
Detection of VacA in mouse tissue. Two weeks after surgical implantation of intragastric catheters, C57BL/6 mice were infused with saline or VacA (500 nM in saline) for, as indicated, either 3 days (target gastric capacity filled to 95%; referred to as short-term exposure), or 30 days (target gastric capacity filled to 50%; referred to as long-term exposure). Animals were immediately euthanized upon completion of each infusion, and tissues were collected, fixed, and paraffin embedded. Sections of the stomach were cut to 5 μm in thickness and stained for IHF imaging, using rabbit anti-VacA antibodies and Alexa Fluor 488-conjugated secondary antibodies, Alexa Fluor 647-conjugated wheat germ agglutinin (WGA; stains mucus granules of gastric surface mucus-producing cells), and DAPI (which stains nuclei). Images are representative of three independent infusion cohorts (n = 3), each performed in triplicate (9 animals total).

The spatial and temporal dynamics of VacA distribution within the stomach during *Hp* infection are poorly understood. However, we speculated that the dispersal of this secreted, diffusible toxin is likely to mirror the gastric niche colonized by *Hp*. Studies of *Hp* biogeography within the stomachs of infected rodents^61–68^ have revealed partitioning of the bacteria as free-swimming bacteria within the gastric mucus, microcolonies on the surface of gastric tissue, and microcolonies associated with epithelial cells that line the stomach glands. Adopting a strategy of administering VacA by intragastric infusion, was based largely on the premise that the toxin delivered into the stomach lumen would diffuse and interact with gastric tissue and glands prior to emptying of gastric contents. Immunohistofluorescence (IHF) imaging of gastric tissue sections collected from animals infused with VacA, but not from animals infused with the vehicle control, revealed detectable green fluorescence, corresponding to VacA, extending from the luminal side of the epithelium into the gastric glands (Fig. 2). These results support a conclusion that at least a portion of VacA, administered into the stomach lumen of catheterized mice, had accessed the gastric glands within the gastric mucosa. In future studies, we will characterize the bio-distribution and concentration of VacA within discrete locations in the upper GI, including the lumen, antrum, corpus, duodenum, and esophagus.

### Gross gastric damage is not detected in response to infused VacA

**Figure 3:**
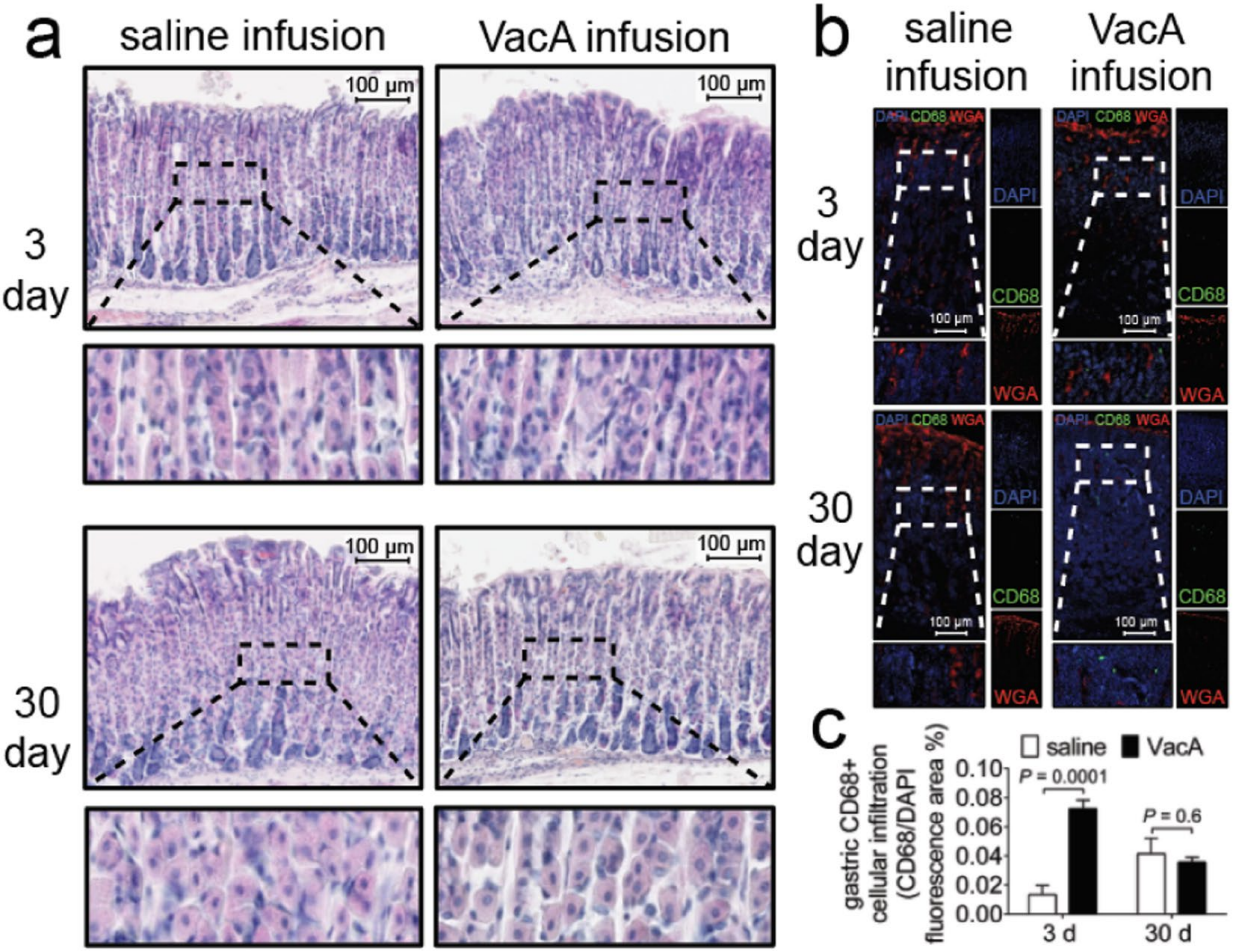
IHF analyses of gastric tissue from VacA-infused mice. Two weeks after surgical implantation of intragastric catheters, C57BL/6 mice were infused with saline or VacA (500 nM in saline), as indicated, for either 3 days (target gastric capacity filled to 95%; referred to as short-term exposure), or 30 days (target gastric capacity filled to 50%; referred to as long-term exposure). Animals were immediately euthanized upon completion of each infusion, and tissues were collected, fixed, and paraffin-embedded. Sections of the stomach were cut to 5 μm in thickness and stained with (**a**) H&E for light microscopy imaging, or, (**b**) for IHF imaging, anti-CD68 antibodies and Alexa Fluor 488-conjugated secondary antibodies, Alexa Fluor 647-conjugated WGA (which stains mucus granules of gastric surface mucus-producing cells), and DAPI (which stains nuclei). Images in (**a**) and (**b**) are representative of those collected from three independent infusion cohorts (n = 3) each performed in triplicate (9 animals total). (**c**) Data from studies described in (**b**) were quantified by comparing green fluorescence emission, corresponding to cell surface CD68+, between gastric tissue collected from mock- and VacA-infused animals. Data were normalized relative to blue fluorescence emission corresponding to nuclei, as a surrogate for the relative number of cells. Error bars represent standard error of the mean of data combined from three independent infusion cohorts (n = 3), each performed in triplicate (9 animals total). Statistical significance was determined at an alpha level of 0.05, using a 2-tailed paired t-test.

Earlier studies indicated that introduction of *Hp* extracts to the stomachs of mice by oral gavage caused damage to gastric tissue^45–49^. To address whether gross damage to gastric tissue also results from VacA administration by intragastric infusion, C57BL/6 mice were infused with either purified VacA (500 nM) or saline vehicle. Unexpectedly, analysis of H&E stained gastric tissue revealed an absence of gross damage associated with mice that had been infused with VacA for either 3 or 30 days (Fig. 3a), similar to tissue collected from mice that had been infused with saline vehicle. The overall glandular structure was not markedly altered, as indicated by a regular alignment of basal-to-apical gastric epithelial cells (Fig. 3a). We did not observe an influx of inflammatory immune cells that would normally accompany tissue damage (Fig. 3a), although using IHF imaging, there was a very modest increase in monocyte-derived cells (CD68^+^ cells) in animals exposed to VacA (Fig. 3b,c), consistent with the idea that VacA alone is not sufficient to elicit the localized inflammatory response characteristic of *Hp*-gastric infection. These data suggest that the substantial alterations and damage that had been previously reported in the gastric tissue of animals with short-term exposure to VacA-containing *Hp* extracts administered by oral gavage^45–49^, were not specifically attributable to VacA.

### VacA-dependent vacuolation of parietal cells

**Figure 4:**
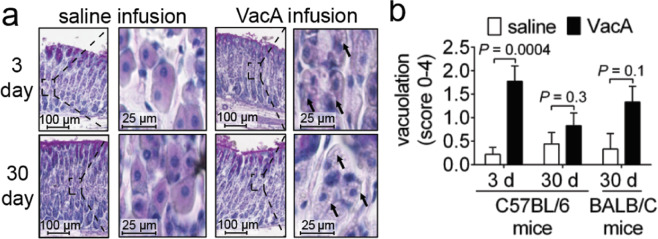
Vacuolation of parietal cells within gastric tissue from VacA-infused animals. Two weeks after surgical implantation of intragastric catheters, C57BL/6 or BALB/C mice were infused with saline or VacA (500 nM in saline), as indicated, for either 3 days (target gastric capacity filled to 95%; referred to as short-term exposure), or 30 days (target gastric capacity filled to 50%; referred to as long-term exposure). Animals were immediately euthanized upon completion of each infusion, and tissues were collected, fixed, and paraffin-embedded. Gastric sections (5 μm in thickness) were prepared from VacA- and saline mock-infused animals (3 different cohorts, each containing 3 animals, for a total of 9 animals from both VacA- and mock-infused animals) by cutting along the greater curvature of the stomach, and then evaluated qualitatively (**a**) and quantitatively (**b**) for relative parietal cell vacuolation. (**a**) Representative light microscopy images of gastric tissue, with black arrows designating examples of intracellular vacuoles. (**b**) Data from the images described in part (**a**) were quantified on a scale from 0–4 (vacuolation in 0% of parietal cells (0), <25% (1), 25–50% (2), 50–75% (3), or >75% (4) of the gastric mucosa). Statistical significance was determined at an alpha level of 0.05, with pairwise comparisons of saline and VacA-infused animals within a single timepoint, using a 2-tailed paired *t*-test. Error bars represent standard error of the mean.

Vacuolation of parietal cells, which was one of the earliest phenotypes associated with VacA function *in vivo*^46,49^, is synonymous with dysfunction, resulting in decreased acid production^69^. Within gastric tissue from mice infused with VacA for 3 days, we observed the presence of vacuolar structures within the cytosol of parietal cells, which were visible as areas of high pallor (Fig. 4a,b). These results are consistent with earlier reports that vacuolar-like structures were reported in damaged gastric tissue of mice that had received *Hp* extracts^46,49^. Parietal cell vacuolation was also clearly visible in tissue prepared from animals infused for 30 days with VacA, although not so evident in animals infused with saline (Fig. 4a**)**. These observations were consistent with higher vacuolation scores within the tissue of animals administered VacA than those infused with saline (Fig. 4b), although the differences were not statistically significant. Notably, several studies have shown that *Hp* can inhibit acid secretion by downregulating H + /K + ATPase expression in parietal cells^70,71^, and that *Hp*-dependent inhibition of acid production in isolated parietal cells has been associated with VacA production^72^. Although we did not examine whether toxin-dependent vacuolation of parietal cells was associated with decreased acid production in these studies, the presence of vacuolar structures can be an indication of secretory dysfunction.

### VacA-dependent reduction in gastric mucus

**Figure 5:**
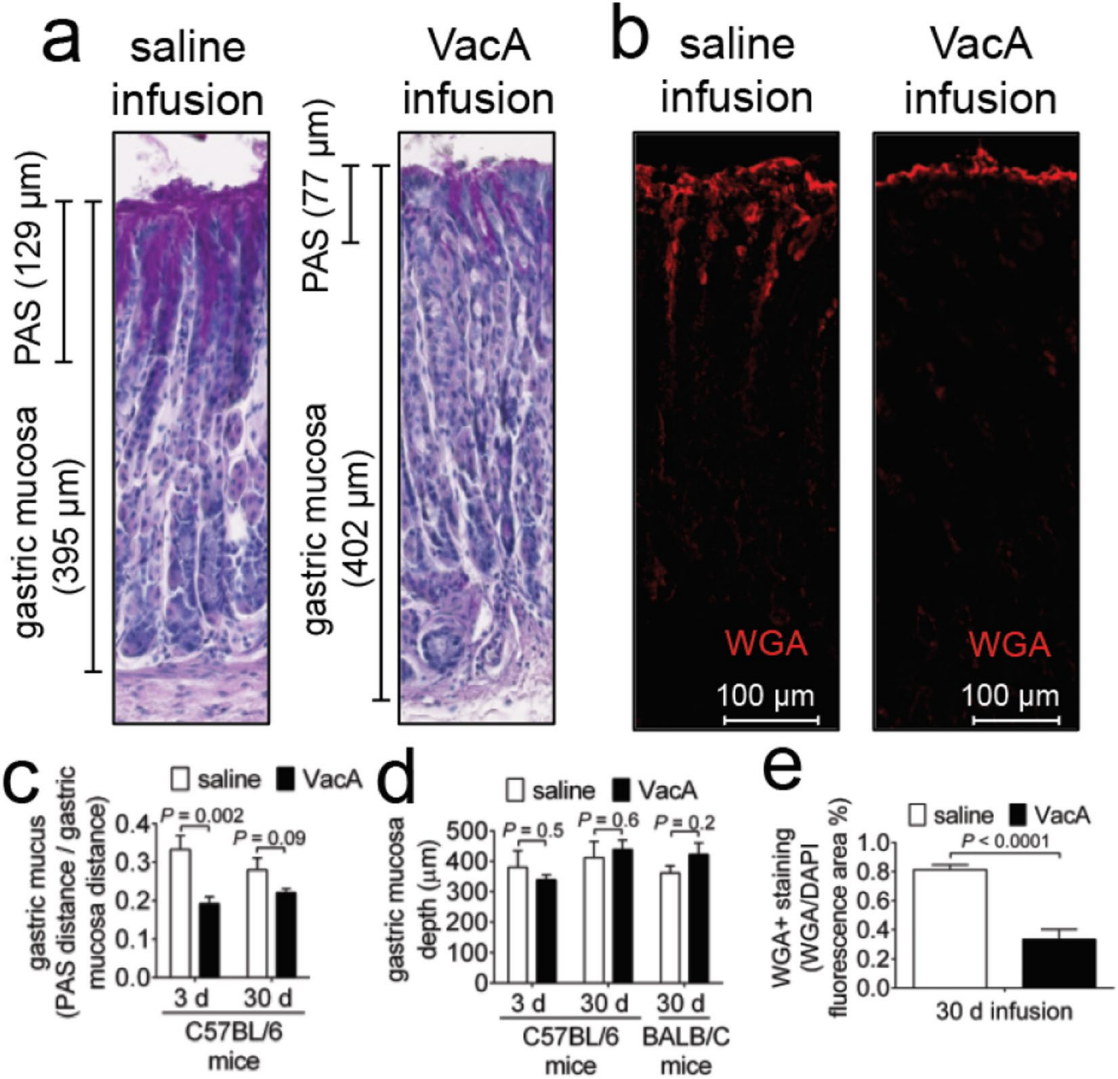
Gastric mucus within gastric tissue from VacA-infused animals. Two weeks after surgical implantation of intragastric catheters, C57BL/6 or BALB/C mice were infused with saline or VacA (500 nM in saline), as indicated, for either 3 days (target gastric capacity filled to 95%; referred to as short-term exposure), or 30 days (target gastric capacity filled to 50%; referred to as long-term exposure). Animals were immediately euthanized upon completion of each infusion, and tissues were collected, fixed, and paraffin-embedded. Gastric sections (5 μm in thickness) were prepared from VacA- and saline mock-infused animals (3 different cohorts, each containing 3 animals, for a total of 9 animals from both VacA- and mock-infused animals), by cutting along the greater curvature of the stomach. The sections were evaluated (**a**) qualitatively for PAS staining, which stains mucus in tissue, by light microscopy imaging, and, (**c**) quantitatively by normalizing the distance of PAS staining from the apical surface of each section relative to (**d**) the total apical-to-basolateral mucosa depth. In addition, tissue sections were stained with Alexa Fluor 647-conjugated WGA, and evaluated (**b**) qualitatively by confocal microscopy for red fluorescence, corresponding to glycoconjugates as a surrogate for mucus, and (**e**) quantitatively by measuring and normalizing the red fluorescence corresponding to the WGA-stained glycoconjugates normalized to blue DAPI fluorescence, corresponding to nuclei, as a surrogate for relative cells in each image. Statistical significance was determined at an alpha level of 0.05, with pairwise comparisons of saline and VacA infused animals within a single timepoint, using a 2-tailed paired *t*-test. Error bars correspond to standard error of the mean.

Another important secretory function associated with gastric glands is the generation of gastric mucus, which is secreted by neck cells in the apical portion of the mucosa. In addition to shielding the surface of the gastric epithelium from the degradative stomach lumen, the thick gastric mucus likely functions as a barrier to *Hp* infection by inhibiting *Hp* penetration into the deeper recesses of the gastric glands, which would be expected to serve as a protective colonization niche. To evaluate if exposure to VacA affects the gastric mucus layer, stomach tissue from C57BL/6 mice infused with either purified VacA (500 nM) or saline vehicle was stained red with Periodic Acid Schiff (PAS) for detection of mucopolysaccharides, neutral mucins, and glycoproteins^73^. In order to control for variability between tissue section thickness, the amount of PAS staining material was normalized to the depth of the gastric mucosa, measured as the distance between the luminal and basal sides of the tissue slice. These studies revealed visibly (Fig. 5a) and quantitatively (Fig. 5b–d) less PAS-staining in gastric tissues collected from mice that had been infused with VacA, as compared to mice infused with saline vehicle.

To further validate our findings, gastric tissue sections that had been incubated with Alexa Fluor 647-labeled wheat germ agglutinin (WGA), which stains mucus granules of gastric surface mucus-producing cells^74^, were examined by confocal microscopy. Although tissue sections of approximately equivalent depth were analyzed, the WGA signal was normalized to the DAPI signal as an internal staining control. These experiments revealed visibly (Fig. 5b) and quantitatively (Fig. 5e) less fluorescence signal, corresponding to WGA, within gastric tissue from mice infused with VacA than animals infused with vehicle control. These data are consistent with an interpretation that VacA is sufficient to cause reduction of the gastric mucus layer, and suggest that VacA may modulate the function of mucus-producing neck cells. Indeed, *in vitro* studies have demonstrated that infection with VacA-producing strains of *Hp* results in the inhibition of mucin synthesis and secretion^75^.

### VacA-dependent *in vivo* gastric alterations extend beyond C57BL/6 mice

All of the studies described above were conducted using C57BL/6 mice. To evaluate whether VacA-dependent alterations in the stomachs of infused animals extend beyond C57BL/6 mice, we examined the effects of short- and long-term VacA exposure in BALB/c mice, which have also been used to investigate *Hp* infection biology^53,76^. Similar to results described above for experiments conducted with C57BL/6 mice, intragastric infusion of purified VacA to BALB/c mice for either 3 or 30 days revealed the absence of gross damage or infiltration of inflammatory immune cells within gastric tissue (Fig. 3). Moreover, VacA-dependent vacuolation of parietal cells was visible in BALB/c mice (Fig. 4b), similar to that observed in C57BL/6 mice.

### Long-term exposure to VacA does not result in detectable intestinal pathology

**Figure 6:**
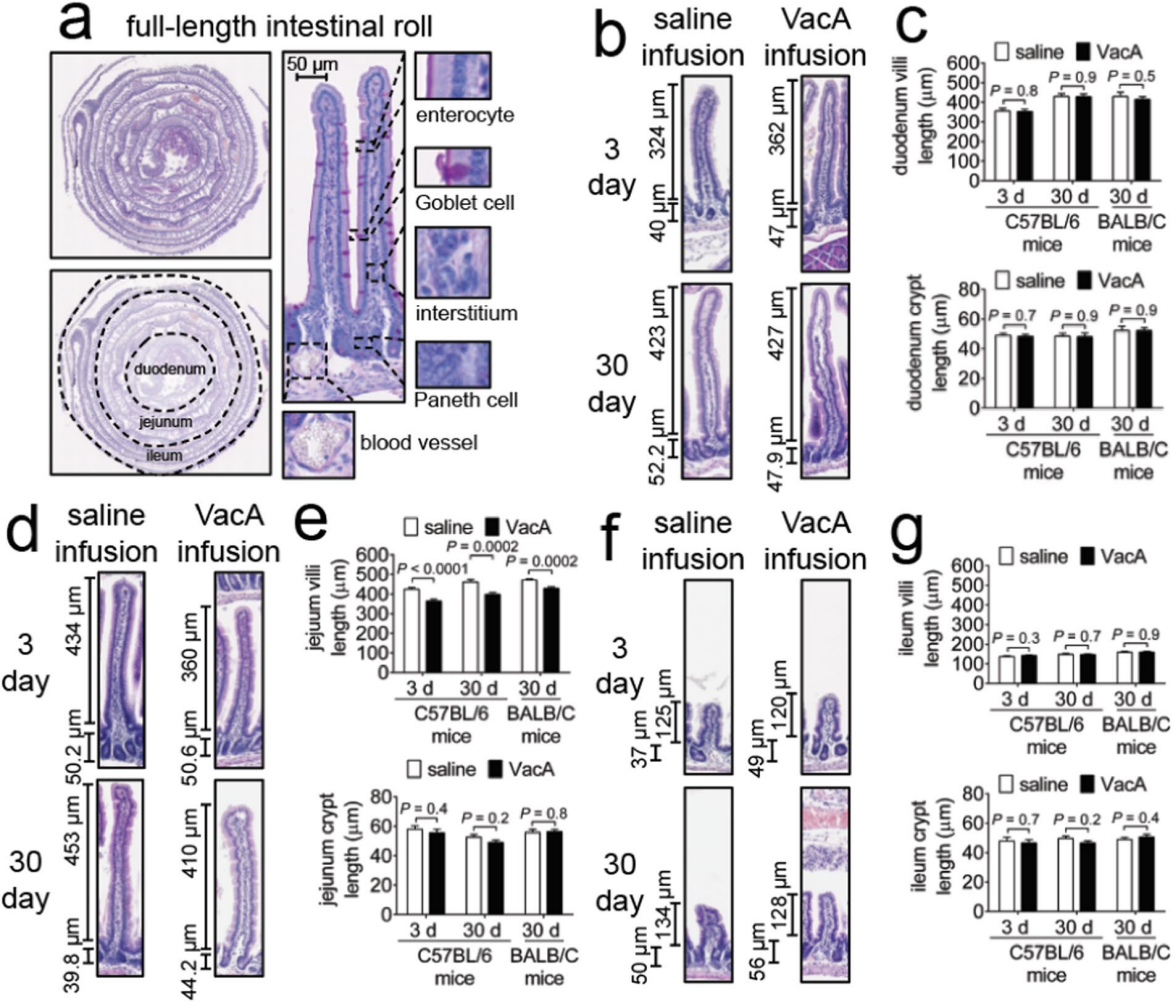
Intestinal histology from VacA- and mock-infused mice. Two weeks after surgical implantation of intragastric catheters, C57BL/6 or BALB/C mice were infused with either saline or VacA (500 nM in saline) for either 3 days (target gastric capacity filled to 95%; referred to as short-term exposure), or, 30 days (target gastric capacity filled to 50%; referred to as long-term exposure). Animals were immediately euthanized upon completion of each infusion, and tissues were collected, fixed, and paraffin-embedded. Transverse sections (short-axis) of rolled intestines were cut to 10 μm in thickness and stained with hematoxylin and eosin (H&E), with all regions of intestine evaluated, as illustrated in (**a**). The (**b,c**) duodenum, (**d,e**) jejunum, and (**f,g**) ileum were (**b,d,f**) imaged and (**c,e,g**) quantified for villi and crypt length by digital measurement. Images are representative of three independent infusion cohorts (n = 3) each performed in triplicate (9 animals total). Statistical significance was determined at an alpha level of 0.05 with pairwise comparisons of saline and VacA-infused animals within a single timepoint with a 2-tailed paired t-test. Error bars correspond to standard error of the mean.

Because gastric contents are emptied from the stomach into the small intestine, we speculated that a portion of infused VacA might enter the intestinal tract during gastric emptying. To evaluate potential “downstream” consequences of VacA, we histologically analyzed entire intestinal tracts, prepared as “Swiss-Rolls”^77^ (Fig. 6a), from C57BL/6 or BALB/C mice that had been infused with either purified VacA (500 nM) or saline vehicle. Comparative analyses of H&E stained tissue, collected from animals exposed to either VacA or saline solution, revealed that there were not differences in villus and crypt lengths within the duodenum (Fig. 6b,c) and ileum (Fig. 6f,g). Within the jejunum, villi lengths were modestly (but significantly) shorter in tissue collected from C57BL/6 mice and BALB/c mice infused with VacA, relative to animals infused with saline solution (Fig. 6d,e). Villus atrophy has been associated with a variety of conditions, including infections with rotavirus^78^, HIV and microsporidia^79^, and *Giardia*^80^. To our knowledge, *Hp*-associated intestinal villus shortening has not been previously reported, and it’s not clear whether the observed phenomenon is a direct or systemic host response to the action of infused VacA.

### Extended, uninterrupted exposure to VacA does not result in detectable esophageal pathology

Although mice and other rodents lack an emetic reflex due to both anatomic (poor diaphragm musculature) and neurogenic (decreased phrenic nerve activity) restrictions^81^, we were concerned that potential overfilling of the stomach during infusion might result in retrograde leakage of toxin from the stomach back into the esophagus, possibly resulting in esophageal damage. However, analysis of H&E stained esophageal tissue from animals infused with VacA indicated that the esophagi did not appear distended, the muscularis mucosa was intact and not enlarged, and gross abnormalities were not detected within the esophageal serosa (Fig. S5). The stratified squamous epithelium was the expected 5–7 layers thick, and, an appropriate layer of keratinized surface material was observed. The absence of observable alterations in esophageal tissue suggest that retrograde spillover of toxin from the stomach into the esophagus was minimal or absent.

### Intragastric VacA infusion does not result in extra-gastric pathology within liver, kidney, pancreas, or lungs

Microscopic imaging of H&E stained tissue from livers of C57BL/6 mice revealed that infusion of VacA (500 nM) for either 3 or 30 days did not result in detectable structural changes or pathology to the hepatic architecture (Fig. S6a). Visible differences were also not detected between kidney, pancreatic, or lung tissues collected from animals administered either VacA or saline vehicle (Fig. S6b–e).

### Intragastric administration of VacA accompanies increased splenic activity

**Figure 7:**
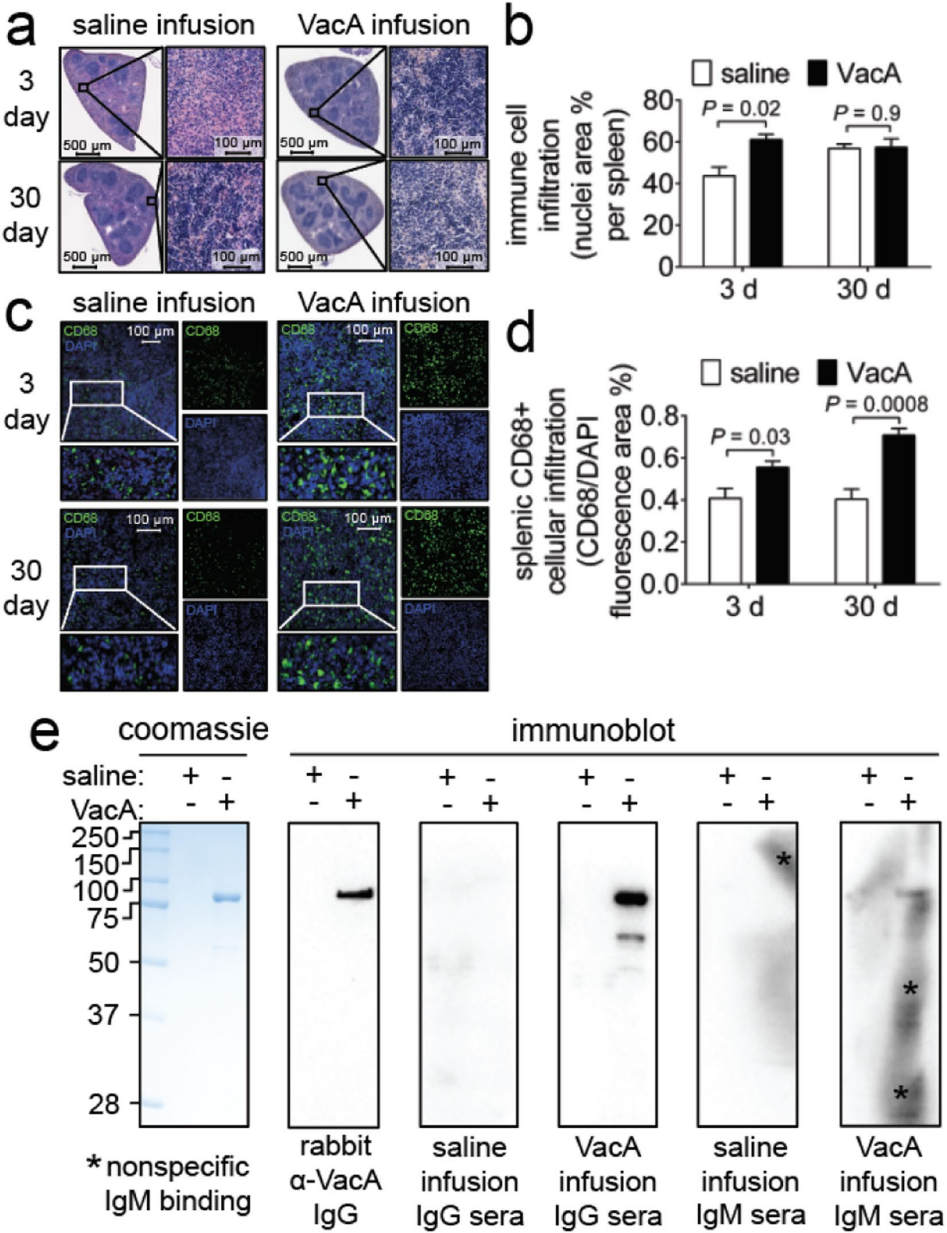
Immune response. Two weeks after surgical implantation of intragastric catheters, C57BL/6 mice were infused with either saline or VacA (500 nM in saline) for either 3 days (target gastric capacity filled to 95%; referred to as short-term exposure), or, 30 days (target gastric capacity filled to 50%; referred to as long-term exposure). Animals were immediately euthanized upon completion of each infusion, and tissues were collected, fixed, and paraffin-embedded. Sections of the (**a,c**) spleen were cut to 5 μm in thickness and stained with (**a**) hematoxylin and eosin (H&E) or (**c**) stained for CD68 using indirect immunofluorescence with Alexa-488 conjugated secondary antibodies, and, DAPI which stains nuclei as a surrogate for cell number, for IHF imaging. Immune cell infiltration into the (**b**) splenic parenchyma was quantified by (**b**) digitally measuring the percentage of the spleen stained with nuclei, or, (**d**) the area of CD68 fluorescence area relative to the area of DAPI staining. (**e**) Purified VacA was immunoblotted using the sera from either 30 day saline or VacA infused mice as a primary antibody, with either anti-mouse IgG or IgM as a secondary antibody. Asterisks indicate nonspecific binding of the IgM antibody. Images are representative of three independent infusion cohorts (n = 3) each performed in triplicate. Statistical significance was determined at an alpha level of 0.05 with pairwise comparisons of saline and VacA infused animals within a single timepoint with a 2-tailed paired t-test. Error bars represent standard error of the mean.

Microscopic imaging of H&E stained splenic tissue revealed that gastric infusion of VacA (500 nM) resulted in an expansion of lymphocytes, as indicated by increased presence of dark staining nuclei in the splenic white pulp (Fig. 7a,b). Within animals administered saline, we speculate that the increased splenic immune cell infiltrate at 30 days, relative to 3 days, reflect normal immune system maturation as mice age. IHF imaging revealed expansion of CD68 + cells, indicating an influx of monocytes and/or monocytic-derived cells (Fig. 7c,d).

### Intragastric VacA infusion stimulates a humoral response

To evaluate whether intragastric infusion of VacA induced an adaptive humoral response, serum was collected from C57BL/6 mice that had been administered either VacA (500 nM) or the saline vehicle control. These studies revealed detectable IgG and IgM antibodies that were immuno-reactive against purified VacA in the sera of 7 of the 9 animals that had been infused with purified toxin for 30-days (but not 3 days) (Fig. 7e). These findings indicated that intragastric infusion of purified VacA resulted in a humoral response in most of the animals.

## Discussion

Molecular genetic approaches have been transformative for establishing the importance of specific microbial factors for pathogen virulence. However, unmasking the exact functional contributions of individual virulence factors to the colonization, survival, and dissemination of microbes during *in vivo* infection remains a challenge in the field of bacterial pathogenesis. This is especially true for secreted and diffusible bacterial exotoxins that can intoxicate host cells both proximal and distal to the immediate site of bacterial colonization within the host^82^. The most direct approach for experimentally evaluating the role of secreted bacterial toxins is to administer the toxin directly to the host, in the absence of infection. However, *in vivo* administration of toxins cannot always faithfully replicate infection conditions. For example, intestinal loop models used to study enteric toxins lack the normal motility and circulatory functions of the intestine^83^. Intravenous administration of toxins may not always accurately reflect the toxin dispersal and bio-distribution routes that normally occur during infection^84^. Finally, the use of single-dose toxin administration, for studying the outcome of acute intoxication, may not provide insights into the consequences of extended toxin exposure during chronic infection.

Here we describe the efficacy of long-term, uninterrupted gastric infusion for the administration of purified VacA into the stomach lumen of mice for 3 or 30 days. *In vivo* infusion of bacterial toxins has been previously employed, perhaps most frequently for the administration of toxin-based therapeutics^85–87^. To experimentally examine the effects of acute toxin exposure in the absence of infection, investigators have used intraperitoneal^88^, intravenous^89^, and intestinal infusion^90^, usually for a period of several hours or less. In contrast, the 30-day infusion period described here provides a more realistic opportunity to study the consequences of protracted exposure to VacA. To our knowledge, intra-gastric infusion has not been previously used to introduce bacterial toxins into the gastrointestinal tract.

An important premise of administering VacA by infusion into the stomach lumen was that the toxin must diffuse and interact with gastric epithelial tissue. Although the bioavailability of VacA during *Hp* gastric infection *in vivo* remains poorly understood, the distribution of VacA within the stomach should reflect the complex “biogeography” of *Hp* at and within gastric glands of infected mice. Congruent with this prediction, we detected VacA associated with the surface epithelium lining the stomach glands of the apical mucosa (Fig. 2), validating that VacA, when infused into the stomach lumen, navigates the thick mucus layer to access the gastric epithelium. Future work will be required to fully characterize the repertoire of cell types within individual glands that are targeted by VacA. Notably, a recent study reported the low-level and transient association of VacA with myeloid cells in mice administered toxin by oral gavage, suggesting that at least a portion of VacA within the gastric mucosa may access the lamina propria^16^. These findings are consistent with a large body of work indicating that VacA interacts with and modulates both epithelial and non-epithelial cells^9,10,91^.

The bioavailability of VacA within the gastric environment during *Hp* infection is poorly understood, but is likely shaped by several factors, including regulation of VacA expression, and secretion of mature toxin to access the extracellular environment during infection. Several studies have indicated that high salt concentrations within the extracellular environment increase both transcription of *vacA*^92^ as well the levels of VacA within the exoproteome of *Hp*^93^, a finding that may be germane to the association between high salt diets and increased stomach cancer risk. In addition, exotoxin diffusion is increasingly being recognized as an important determinant of concentration-dependent modulation of the infection microenvironment^94^. The findings here that infused VacA interacts with gastric tissue and modulates several gastric functions previously associated with *Hp* infection, provides the framework for future studies to establish dose-dependent and cell-type specific alterations attributable uniquely to VacA. Indeed, we are particularly interested in examining potential VacA-dependent gastric alterations at toxin concentrations lower than those at which cell death is observed. In our *in vitro* studies, we have noted VacA dependent alterations, including vacuolation, mTORC1 inhibition, and autophagy induction at concentrations 10–100 fold-lower than those required for cell death. This work will ultimately be most informative when complemented by parallel efforts to define the *in vivo* distribution and availability of VacA during *Hp* infection.

Strikingly, our studies here did not reveal the gross tissue damage that had been earlier reported in mice that had received, by oral gavage, boluses of VacA-containing *Hp* extracts^45–47,49^ We speculate that the toxin preparations used in earlier studies may have included pro-inflammatory components not present in our VacA preparations, such as lipopolysaccharide, urease, and the neutrophil activating protein (NAP). Even in the absence of tissue damage, our data indicated that infused VacA was sufficient to induce a humoral immune response, consistent with previous reports that oral immunization of mice with purified VacA protected animals from infection with cytotoxin producing strains of *Hp*^45,95^.

While gross damage was not detected within VacA-infused animals, several gastric alterations previously associated with *Hp* infection were detected, including a significant reduction in the gastric mucus layer (Fig. 5). Notably, a decrease in gastric mucus in animals infected with *Hp*^64,96^ or orally gavaged with *Hp* extracts^46^ had been earlier reported. Because our studies revealed infused VacA associated with glands at the apical side of the gastric mucosa, which is enriched with mucus-secreting neck cells, we conjecture that VacA may directly modulate mucus production by intoxication of these cells. A prominent consequence of VacA cellular intoxication is an overall reduction in host cell biosynthetic metabolism, resulting from toxin-mediated mitochondrial dysfunction^20,21,31,36,97,98^. The resulting depletion in cellular energy is sensed by the mammalian target of rapamycin complex 1 (mTORC1)^21^, resulting in a global cellular shift to catabolic metabolism. Because mTORC1 directly regulates protein translation, we speculate that pruning of the gastric mucus layer during *Hp* infection may result from VacA-dependent attenuation of mucin biosynthesis, which would be consistent with previous work suggesting that mucin synthesis is affected during *Hp* infection, rather than mucin secretion and release into the extracellular milieu^99^. Although the ramifications of VacA-dependent inhibition of gastric mucus are not clearly understood, one possibility is that thinning of the mucus layer may promote deeper penetration of bacteria into the gastric glands as a privileged colonization site.

We also observed parietal cell vacuolation exclusively in animals infused with purified VacA. Reduction in stomach acidity, or hypochlorhydria, is a hallmark of chronic human infection with toxigenic strains of *Hp*, and is associated with *Hp* gastric disease^100,101^, and eradication of *Hp* infection restores normal parietal cell ultrastructure and function^102,103^. In animal models, VacA-dependent parietal cell dysfunction and loss of acid production has been reported in isolated gastric glands from mice^102^ and guinea pigs^104^. While the implications of VacA targeting of parietal cells are not fully understood, reduced acidity of the stomach is likely to promote chronic gastric infection of *Hp*, which are not acidophilic.

While the studies described here focused on VacA-dependent alterations of the gastric epithelium, future work will also examine immune cell modulation in the stomachs of mice administered toxin by intragastric infusion. *In vitro* studies have revealed that VacA targets and alters the function of immune cells, and in particular myeloid cells and T lymphocytes^105^. VacA-mediated Interference with T cell activation occurs by both channel-dependent and -independent mechanisms^106^. Future studies to evaluate whether these two independent mechanisms also exist *in vivo* will be facilitated by gastric infusion of mutant forms of VacA lacking membrane channel activity.

In summary, results from these studies support the efficacy of intragastric infusion for studying the effects of extended, uninterrupted administration of VacA into the stomachs of mice. Notably, VacA-dependent gastric alterations were manifested early (after only 3 days of infusion), and were generally maintained, as some effects remained after 30 days of infusion. Extended studies throughout the different life stages of experimental animals (i.e. adolescence, sexually mature, socially mature, aged, etc.), will be required to fully understand the dynamics, longevity, and reversibility of VacA-dependent modulation of the gastric environment. Notably, intragastric infusion is scalable to accommodate higher numbers of animals, as well as other small rodent species. In addition, the programmable interface bestows a high degree of flexibility for examining a broad array of alterations resulting from VacA exposure *in vivo*, including dose-dependent, toxin-specific modifications to host cell transcriptomes and proteomes, as well as cell-specific responses to chronic VacA exposure.

## Materials and Methods

### Animal care

All experiments involving the use of live vertebrate animals were conducted in accordance with the National Institutes of Health Guide for the Care and Use of Laboratory Animals, and, with the approval of the University of Illinois at Urbana-Champaign Institutional Animal Care and Use Committee (Protocol #15239). C57BL/6 mice, bred in-house, and BALB/C mice (purchased from Jackson Laboratory, Bar Harbor ME), were housed in a climate-controlled (23 °C, 55% humidity), animal care unit with a 12 h:12 h light:dark cycle. Animals were fed a standard commercial rodent diet (Envigo, Indianapolis, IN) and allowed access to food and water *ab libitum*. C57BL/6 mice were sibling-mated in trios (2 females and 1 male), and the pups were weaned at 22 days of age. Experimental procedures were commenced when animals were 5–6 weeks old. After surgical placement of the intragastric catheter, animals were housed singly for the duration of the studies.

### Analgesia

Mice were administered pre-operative analgesia of carprofen (5 mg/kg; Zoetis, NJ), buprenorphine (0.05 mg/kg; Par Pharmaceuticals, NJ), and cefazolin (20 mg/kg; West-Ward Pharmaceutical, TN). Animals were also administered post-operative analgesia of buprenorphine (0.05 mg/kg), 2 and 24 h after surgery. All medications were dispensed subcutaneously on the right flank of each animal.

### Surgical placement of intragastric catheter

Following the induction and maintenance of anesthesia with isoflurane (5% induction, 1.5% maintenance, in 100% O_2_, Henry Schein, NY), the ventral abdomen, left thorax, and dorsal cervical area of mice were aseptically prepared for surgery. To each animal in dorsal recumbency, a midline celiotomy (1 cm) was performed just caudal to the xiphoid process. The stomach was gently exteriorized from the abdominal cavity using DeBakey forceps. Pre-sterilized polyurethane intragastric catheters were custom-ordered (8 cm long, 0.6 mm inner diameter, 1.0 mm outer diameter; Norfolk Medical, Access Technologies, Skokie Illinois) with an unattached 23 G, 0.5” blunt Luer needle, and were passed through a 0.75 cm diameter polyethylene terephthalate (Dacron) suture disk, and positioned 0.5 cm from the distal end of the slit valves. The catheters were inserted into the greater curvature of the forestomach by puncturing the gastric serosa with a 29 G 3.5” spinal needle (BD Biosciences, NJ) within the catheter and were adhered to the gastric serosa by application of a single drop (~10 µL) of sterile internal cyanoacrylate tissue adhesive (GLUture, Norfolk Medical, Skokie Illinois) to the suture disk. The free-end of the intragastric catheter was passed through and then adhered to the body wall with a 14 G I.V. catheter (BD Biosciences) using sterile cyanoacrylate tissue adhesive applied onto the suture disc. Subsequent to exteriorization of the free end of the intragastric catheter from the left subcutaneous space to the dorsal cervical region, all incisions were closed with 5–0polyclactin suture on a taper point needle (Ethicon, Sommerville, NJ). The external intragastric catheter was connected to a Luer port, capped, and secured to the dorsal skin by 2–3 simple interrupted sutures.

### Post-surgical monitoring

Mice were monitored for body weight and pain every 2 h for the first 12 h following surgery, and then every 12 h for the ensuing 2–3 weeks. Pain was assessed on a 0–10 scale (0 = no pain, 10 = severe pain) by assessing for normal mouse behavior (movement, grooming, piloerection, posture, eye position), and surgery-related stressors (dehiscence, inflammation, infection).

### Purification of VacA

*Helicobacter pylori (Hp)* 60190 (*cag* PAI^+^, *vacA* s1/m1; ATCC 49503) was cultured in bisulfite/sulfite-free *Brucella* (BSFB) broth (4 mL, 0.5% NaCl, 0.2% β-cyclodextrin, 0.1% dextrose (all from Sigma-Aldrich, St. Louis, MO), and, 1% peptone, 1% tryptone, and 0.2% yeast extract (all from BD Bacto, NJ), overlaid on 2% agar plates made of Ham’s F-12 medium (Sigma-Aldrich) supplemented with 5% fetal bovine serum (FBS) (Sigma-Aldrich)), and incubated in a microaerophilic environment (10% O_2_, 5% CO_2_). After 48 h, the liquid culture phase was collected as a starter culture, and diluted 1:100 in fresh BSFB (final 2 L) supplemented with 5 µg/mL vancomycin (Sigma-Aldrich), and incubated on a shaker platform in a humidified, microaerophilic (10% O_2_, 5% CO_2_), 37 °C incubator. After 48 h, *Hp* cultures were centrifuged (5,000 × *g*, 4 °C, 30 min), the supernatants were collected, and VacA was precipitated by adding solid ammonium sulfate (Fisher Scientific) to 90% saturation (662 g/L) at 4 °C with constant stirring. After 4 h, the precipitated material was collected by centrifugation (5,000 × *g*, 4 °C, 30 min) and dissolved in “wash buffer” (10 mM Na_2_HPO_4_, pH 7.0; Sigma-Aldrich), and then dialyzed at 4 °C in the wash buffer (200 times the volume of the precipitate solution), with four buffer changes spanning 12 h, using 50 kDa molecular weight cutoff (MWCO) dialysis tubing (Spectra/Por 6 RC, Spectrum Labs, City, ST). The dialyzed solution was cleared of any remaining insoluble material by centrifugation (5,000 × *g*, 4 °C, 30 min), and the supernatant was further cleared by filtration using a 0.22 μM MWCO filtration funnel (Stericup, EMD Millipore, City, ST). The VacA-containing soluble fraction was loaded onto an anion exchange column (DEAE Sephacel, GE healthcare, City, ST) pre-equilibrated with wash buffer. After washing the column with 3 bed volumes of wash buffer, VacA was eluted from the resin with wash buffer supplemented with 0.2 M NaCl and collected in 1 mL fractions. The purity of VacA within each fraction was assessed by SDS-PAGE and Coomassie Staining (G-250 Coomassie Brilliant Blue; Sigma-Aldrich). Fractions containing VacA without additional visible protein bands were combined and dialyzed for 12 h (with 4 buffer changes) at 4 °C in saline (0.9% NaCl, at 200 times the volume of the purified VacA), using 50 kDa MWCO dialysis tubing. Following dialysis, purified VacA was filter-sterilized using a 0.22 μm pore syringe filter, aliquoted into single use vials, and stored at −20 °C. The total protein concentration was determined using the bicinchoninic acid (BCA) assay (Thermo Fisher Scientific). The presence of VacA was confirmed by immunoblot analysis using rabbit VacA antiserum that was generated in-house with New Zealand white rabbits, as described below under “Polyclonal VacA antibody production”.

### Acid activation of VacA

Immediately prior to infusion, VacA was activated by mixing 10% (v/v) HCl (0.3 M) to purified toxin, and then incubated at 37 °C, as described^107^. After 30 min, the solution was neutralized by adding NaOH (0.3 M) at an equivalent volume as the HCl added, and diluted to the final infusion concentration with sterile saline (0.9% NaCl).

### Polyclonal VacA antibody production

Animal usage was conducted in accordance with the National Institutes of Health Guide for the Care and Use of Laboratory Animals, and, was approved by the IACUC at the University of Illinois, Urbana-Champaign (Protocol #15019). Two female New Zealand white rabbits (10–12 weeks old; KBL (NZW) Charles River, Wilmington MA) were co-housed within a climate-controlled (23 °C, 55% humidity) University of Illinois animal care unit with a 12 h:12 h light:dark cycle. Animals were fed a standard commercial rabbit diet (Envigo, Indianapolis, IN) and allowed access to food and water *ab libitum*. For immunization and blood collection, animals were sedated with acepromazine (1 mg/kg). Animals were immunized with purified VacA (1 mL, 0.5 mg/mL), in a 1:1 v/v emulsion with the adjuvant Titer Max Gold (Sigma Aldrich), by subcutaneous injection at 5 separate sites on the haunches of the animals. Three secondary immunizations, consisting of purified VacA (1 mL, 0.5 mg/mL) in a 1:1 v/v emulsion with incomplete Freund’s Adjuvant (Thermo Fisher), were given at 2-week intervals. Three booster immunizations, consisting of purified VacA (1 mL, 0.5 mg/mL) in a 1:1 v/v emulsion with incomplete Freund’s Adjuvant, were given at 3-week intervals, beginning 3 weeks after beginning serum collections. Following a titer test bleed conducted 5 weeks after the primary immunization, production bleeds were conducted every 2 weeks. Production bleeds of no more than 1% of the animal’s weight of whole blood were collected from the central ear artery into serum-separating vacutainers (BD Biosciences, NJ). After 19 weeks, a terminal cardiac blood collection was performed while animals were anesthetized under ketamine (Med Vet International, Mettawa, IL) (50 mg/kg) and xylazine (Sigma Aldrich) (10 mg/kg) anesthesia, followed by euthanasia with pentobarbital (100 mg/kg). Whole blood was incubated at 4 °C for 24 h, then centrifuged (1,500 *xg*) at 4 °C. After 15 min of centrifugation, serum supernatants were pooled, and sodium azide (Sigma-Aldrich) was added to a final concentration of 0.05%, and aliquots were stored at −20 °C. Antibody titers were evaluated by immunoblotting purified VacA with the anti-VacA sera.

### SDS-PAGE and immunoblotting

Purified VacA protein was denatured using 2X Laemmli sample buffer (Bio-Rad, CA) supplemented with 10% β–mercaptoethanol (Sigma-Aldrich), boiled, and resolved using SDS-PAGE. VacA was visualized after staining with Coomassie Brilliant Blue. For immunoblotting, proteins were electro-transferred to polyvinylidene fluoride (PVDF, Biorad) membranes, probed with anti-VacA polyclonal primary antibodies and horseradish peroxidase (HRP) conjugated secondary antibodies (Cell Signaling, MA) and visualized by chemiluminescence (SuperSignal West Femto/Pico Maximum Sensitivity Substrate; ThermoFisher). Images were acquired using a ChemiDoc XRS + system (Bio-Rad) and data were processed with ImageLab software (Bio-Rad, version 4.1, NJ).

### Intragastric infusion

Following recovery from surgery, mice were connected to infusion line tubing via the dorsally-placed Luer port (Fig. S2). Infusion solutions were drawn from sterile, 25 mL reservoirs, and delivered to mice through polyurethane and silicone tubing using a peristaltic pump (BT100-2J, Langer Instruments). For these studies, both purified VacA and the vehicle control were acid activated Short-term infusions were conducted for three days, and consisted of an initial bolus administered at 40 μL/min for 5 min, and then afterwards dispensed every 30 min for a duration of 1 min, and at a rate of 40 μL/min. Long-term infusions were conducted for thirty days, and consisted of an initial bolus administered at 100 μL/min for 1 min, and then dispensed afterwards every 45 min for a duration of 1 min, and at a rate of 50 μL/min. Every 24 h during an infusion series, immediately following a scheduled re-infusion administration, the system was paused, the lines were disconnected, and residual toxin was drawn out of the lines and reservoir using positive pressure. The reservoirs were filled, the lines were primed with fresh toxin, and the system was restarted (<5 min exchange time). At the completion of each infusion period, the infusion system was stopped and the lines were disconnected, and animals were placed in a separate clean cage for CO_2_ asphyxiation, with death confirmed by cervical dislocation.

### Detection of VacA antibodies within mouse sera

Following immunoblotting of purified VacA, full-lane length PVDF membranes were incubated in a 1:1000 dilution of sera collected from infusion animals overnight at 4 °C. After primary antibody incubation, the membranes were washed with 0.1% TBS-T, and incubated in either anti-mouse IgG or IgM, conjugated to HRP (IgG-HRP: Cell Signaling Technology; IgM-HRP: Thermo Fisher). Full blots were visualized simultaneously by incubating each blot in equal concentrations of chemiluminescence substrate (SuperSignal West Femto/Pico Maximum Sensitivity Substrate; ThermoFisher). Images in each experimental replicate were acquired together under identical exposure conditions using a ChemiDoc XRS + system (Bio-Rad), and data were processed with ImageLab software (Bio-Rad, version 4.1, NJ).

### Paraffin embedded tissue processing

The stomach, intestines, esophagus, spleen, and liver were collected from euthanized animals and immediately fixed in Bouin’s fixative (15:5:1 saturated picric acid (Sigma): 40% formalin (Sigma) in 0.1 M phosphate buffer: glacial acetic acid (Fisher Scientific)) at room temperature for 4 h. The intestines were prepared from the proximal duodenum to the distal ileum, using the “Swiss Roll” method^77,108^. Following extensive washing in PBS pH 7.4, all tissues were paraffin embedded, and tissue sections were cut to 5–7 μm thick using a microtome (Leica RM 2255 rotary microtome, Germany), and then applied to microscope slides with a positively charged surface coating (Globe Scientific, NJ). The slides were dried overnight at 37 °C, and stored at room temperature.

### H&E and PAS staining

Paraffin-embedded tissue sections were deparaffinized and stained in Gills 1 hematoxylin (Sigma) and Eosin (Sigma), or PAS (EM Biosciences, PA), and counterstained in Mayer’s hematoxylin (Sigma). Stained slides were mounted in CytoSeal mounting medium (Thermo Fisher), a coverslip was added, and the slides were allowed to dry. Slides were imaged by light microscopy, digitized by scanning (NanoZoomer, Hamamatsu, Japan), and analyzed using the NDP.view2 software (Hamamatsu).

### Fluorescence microscopy

Paraffin-embedded tissue sections were deparaffinized and hydrated with tap water. Heat-induced antigen retrieval was performed by incubating slides in citrate antigen retrieval buffer (10 mM sodium citrate, 0.05% Tween-20, pH 6.0) at 100 °C. After 20 min, the slides were allowed to cool to room temperature, rinsed with Tris buffered saline (TBS; 50 mM Tris-Cl, 150 mM NaCl, pH 7.5), and then permeabilized by incubating the presence of in 0.25% TritonX-100 in TBS at room temperature. After 10 min, the slides were blocked by incubating at room temperature in the presence of 5% bovine serum albumin (BSA) in SeaBlock blocking buffer (Thermo Fisher). After 60 min, the slides were then incubated at 4 °C in the presence of primary antibody (the specific antibody was diluted in 1% BSA in TBS, as indicated for each specific experiment). After overnight incubation, slides were rinsed with TBS and subsequently incubated in Alexa Fluor conjugated secondary antibody diluted in 1% BSA/TBS at room temperature. After 60 min, slides were rinsed with TBS and incubated in DAPI (0.5 µg/ml DAPI in PBS) at room temperature. After 15 min, slides were washed with TBS, mounted in ProLong Gold Antifade Reagent (Thermo Fisher), and sealed with a coverslip. All fluorescently stained tissues were imaged using a Nikon A1 laser confocal microscope. Images were deconvoluted using AutoQuantX, with the fixed point-spread function (PSF) (non-blind) theoretical PSF program, consisting of a total of 20 iterations with 20 intervals and the noise level set to low (2).

### Statistical analysis

All infusions were performed using three independent replicate blocks, with three replicate animals per block. Data were tested for normality using a Shapiro-Wilk normality test. Datasets that failed to reject the null hypothesis (P value > 0.05) were analyzed by a parametric Student’s t-test for pairwise comparisons. Datasets that rejected the null hypothesis (P value < 0.05) were analyzed by a nonparametric Student’s t-test for pairwise comparisons. For pairwise comparisons, significance was determined using a 2-tailed distribution, paired *t* test. For multiple comparisons, significance was determined using a one-way ANOVA with the Tukey’s correction for multiple comparisons. Unless otherwise indicated, statistical significance was signified by an alpha level threshold of 0.05. Error bars represent standard error of the mean (SEM). Graphpad Prism (version 6.01) was used for all statistical analyses.

## Supplementary information

Supplementary Information.
