# Supplementary material for: Chronic in vivo exposure to Helicobacter pylori VacA: Assessing the efficacy of automated and long-term intragastric toxin infusion
Source: Sci Rep. 2020 Jun 9;10:9307. doi: 10.1038/s41598-020-65787-3 (PMC7283276; doi:10.1038/s41598-020-65787-3)
Supplement: Supplementary file 1 — Supplementary Information. [file 41598_2020_65787_MOESM1_ESM.docx]

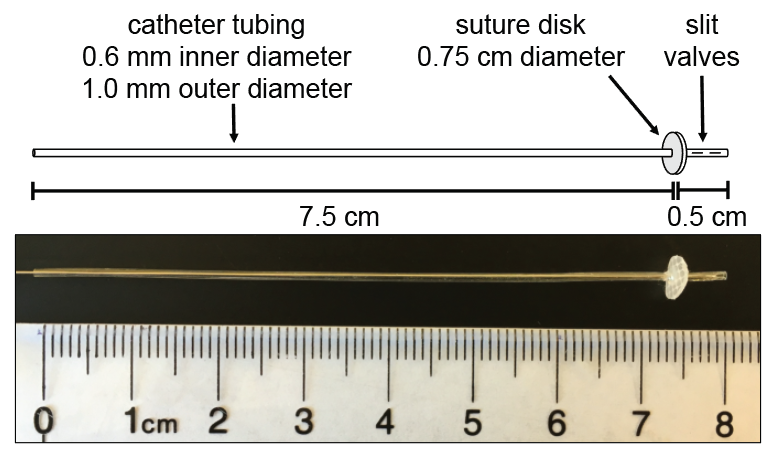
 **Supplementary Figure 1. Intragastric catheter design.** The intragastric catheter was 8 cm in length, with a 0.75 cm diameter suture disk placed 0.5 cm from the distal end and 7.5 cm from the proximal end of the catheter. Slit valves were included on the distal end of the catheter to limit clogging or blockage of the catheter port. The inner diameter of the catheter was 0.6 mm, and the outer diameter was 1.0 mm. The distal end of the catheter was inserted into the stomach, with the proximal end externalized on the back of the animal and attached to the suture disk to secure the stomach to the body wall. Images were created using Adobe Illustrator 2015.1.0.


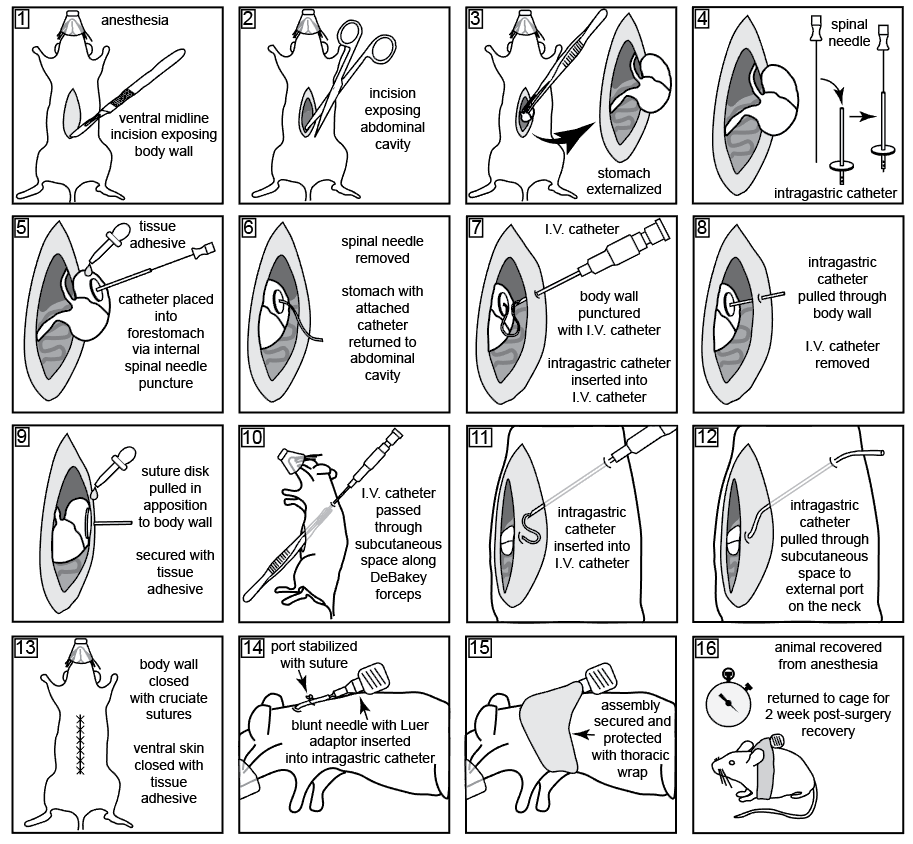
 **Supplementary Figure 2. Surgical placement of intragastric catheter.** (panels 1, 2) After induction of anesthesia, pre-operative medications were administered, a sterile surgical site was prepared, a ventral midline incision was made, and (panel 3) the stomach was exteriorized. (panel 4) A 19 gauge spinal needle was placed within the intragastric catheter, and (panel 5) the catheter was inserted into the forestomach and adhered to the gastric serosa by tissue adhesive, (panel 6) followed by removal of the spinal needle. (panels 7, 8) By use of an intravenous (I.V.) catheter, the intragastric catheter was drawn through the body wall, and (panel 9) adhered to the body wall with tissue adhesive. (panel 10) By use of an I.V. catheter, (panels 11, 12) the intragastric catheter was drawn under the lateral subcutaneous space and exteriorized on the dorsal cervical region (back of the neck). (panel 13) The abdominal incision (ventral body wall) was closed, (panel 14) the external intragastric catheter was connected to a Luer port, and (panel 15) the assembly was secured with a bandage (thoracic wrap). (panel 16) Mice recovered from anesthesia, and were monitored for 2 weeks for post-operative recovery. Images were created using Adobe Illustrator 2015.1.0.


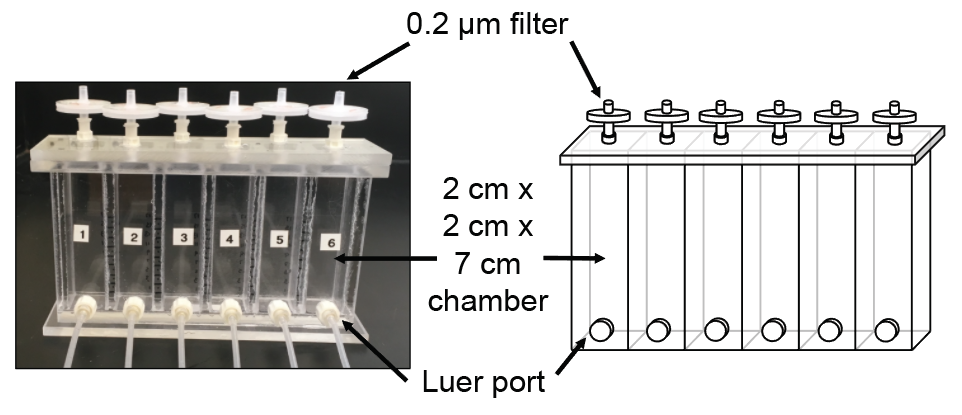
 **Supplementary Figure 3. Infusion reservoir design.** Solutions to be infused into animals were stored in a sterile, six-chambered acrylic reservoir. The opening of each chamber was fitted with a 0.2 μm pore filter to prevent the formation of a vacuum while solution was being infused, while also maintaining the sterility of the infused solution. Each chamber was 2 cm in width, 2 cm in depth, and 7 cm in height, with a total volume of capacity of 28 mL. Infusion lines were connected to each chamber by a Luer port fitted at the bottom of each chamber. Immediately prior to and after each infusion, the inner chamber of the reservoir was sterilized by washing with 70% ethanol, followed by rinses with sterile saline. Three chambers were designated specifically for saline infusion solutions, and three chambers were designated specifically for VacA. Images were created using Adobe Illustrator 2015.1.0.


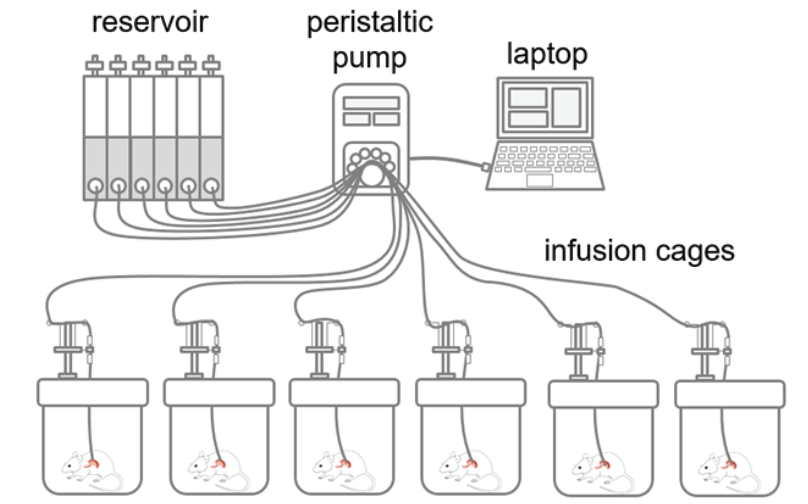
 **Supplementary Figure 4. Intragastric infusion setup.** Mice with implanted intragastric catheters were connected to the intragastric infusion apparatus after a 2 week recovery period. Mice were singly housed and connected to infusion lines by a Luer port on the dorsum (back) of the animal. Infusion lines were connected to a reservoir containing infusion solution. The volume of solution infused into the animals was controlled by a peristaltic pump controlled locally with a laptop computer. Infusions were conducted using cohorts of 6 simultaneously infused animals, consisting of 3 animals infused with VacA (500 nM, in saline solution (0.9% NaCl), and 3 animals infused with the saline vehicle control. Images were created using Adobe Illustrator 2015.1.0.


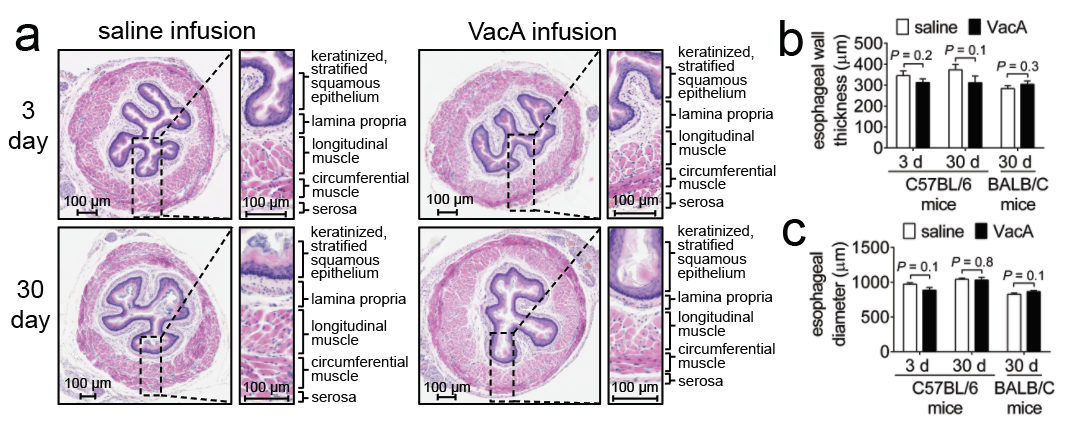


**Supplementary Figure 5. Esophageal histology.** Two weeks after surgical implantation of intragastric catheters, C57BL/6 or BALB/C mice were infused with either saline or VacA (500 nM in saline) for either 3 days (target gastric capacity filled to 95%; referred to as short-term exposure), or, 30 days (target gastric capacity filled to 50%; referred to as long-term exposure). Animals were immediately euthanized upon completion of each infusion, and tissues were collected, fixed, and paraffin-embedded. (a) Transverse sections (short-axis) of the esophagus were cut to 5 μm in thickness and stained with hematoxylin and eosin (H&E). (b) The thickness of the esophageal wall was measured from the apical keratinized, stratified squamous epithelium, to the serosa, at each maximum and minimum thickness of each esophageal fold. (c) The esophageal diameter was measured in three evenly spaced transects. Images are representative of three independent infusion cohorts (n=3), each performed in triplicate (9 animals total). Statistical significance was determined at an alpha of 0.05 level with pairwise comparisons of saline and VacA infused animals within a single timepoint with a 2-tailed paired t-test. Error bars correspond to the standard error of the mean.


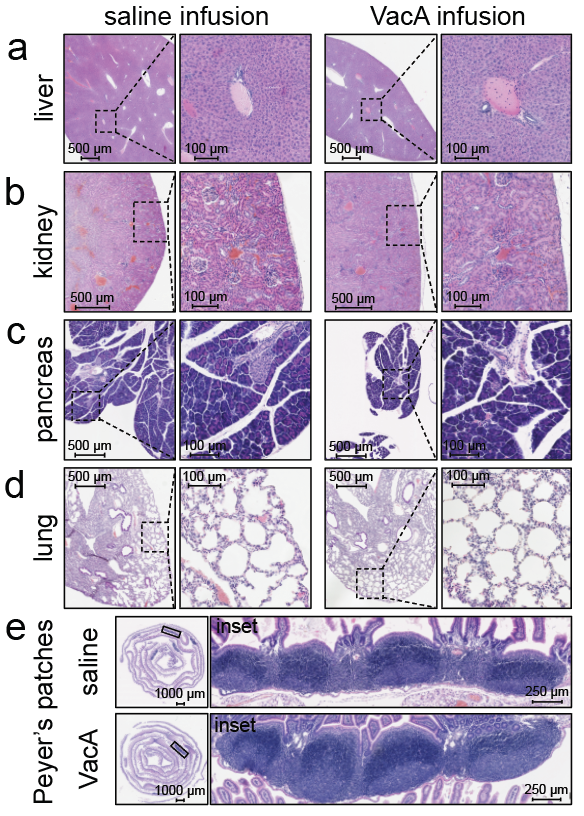


**Supplementary Figure 6. Hepatic, renal, pancreatic, pulmonary, and Peyer’s patch histology.** Two weeks after surgical implantation of intragastric catheters, C57BL/6 mice were infused with either saline or VacA (500 nM in saline) for either 3 days (target gastric capacity filled to 95%; referred to as short-term exposure), or, 30 days (target gastric capacity filled to 50%; referred to as long-term exposure). Animals were immediately euthanized upon completion of each infusion, and tissues were collected, fixed, and paraffin-embedded. Sections of the liver (a) kidney (b), pancreas (c), lung (d) and Peyer’s patches (e) were cut to 10 μm in thickness and stained with hematoxylin and eosin (H&E). Images are representative of three independent infusion cohorts (n=3) each performed in triplicate.
